# Enhancement of tolerance development to morphine in rats prenatally exposed to morphine, methadone, and buprenorphine

**DOI:** 10.1186/1423-0127-17-46

**Published:** 2010-06-07

**Authors:** Yao-Chang Chiang, Tsai-Wei Hung, Cynthia Wei-Sheng Lee, Jia-Ying Yan, Ing-Kang Ho

**Affiliations:** 1 Division of Mental Health & Addiction Medicine, Institute of Population Health Sciences, National Health Research Institutes, 35 Keyan Road, Zhunan, Miaoli County 35053, Taiwan ROC

## Abstract

**Background:**

Abuse of addictive substances is a serious problem that has a significant impact on areas such as health, the economy, and public safety. Heroin use among young women of reproductive age has drawn much attention around the world. However, there is a lack of information on effects of prenatal exposure to opioids on their offspring. In this study, an animal model was established to study effects of prenatal exposure to opioids on offspring.

**Methods:**

Female pregnant Sprague-Dawley rats were sub-grouped to receive (1) vehicle, (2) 2-4 mg/kg morphine (1 mg/kg increment per week), (3) 7 mg/kg methadone, and (4) 3 mg/kg buprenorphine, subcutaneously, once or twice a day from E3 to E20. The experiments were conducted on animals 8-12 weeks old and with body weight between 250 and 350 g.

**Results:**

Results showed that prenatal exposure to buprenorphine caused higher mortality than other tested substance groups. Although we observed a significantly lower increase in body weight in all of the opioid-administered dams, the birth weight of the offspring was not altered in all treated groups. Moreover, no obvious behavioral abnormality or body-weight difference was noted during the growing period (8-12 weeks) in all offspring. When the male offspring received morphine injection twice a day for 4 days, the prenatally opioid-exposed rats more quickly developed a tolerance to morphine (as shown by the tail-flick tests), most notably the prenatally buprenorphine-exposed offspring. However, the tolerance development to methadone or buprenorphine was not different in offspring exposed prenatally to methadone or buprenorphine, respectively, when compared with that of the vehicle controlled group. Similar results were also obtained in the female animals.

**Conclusions:**

Animals prenatally exposed to morphine, methadone, or buprenorphine developed tolerance to morphine faster than their controlled mates. In our animal model, prenatal exposure to buprenorphine also resulted in higher mortality and much less sensitivity to morphine-induced antinociception than prenatal exposure to morphine or methadone. This indicates that buprenorphine in higher doses may not be an ideal maintenance drug for treating pregnant women. This study provides a reference in selecting doses for clinical usage in treating pregnant heroin addicts.

## Background

Opioid drugs are the most effective therapeutic analgesic for chronic pain and cancer pain. Continual use of opioids, however, results in the development of tolerance and dependence. Moreover, widespread abuse of opioids (heroin and/or morphine) causes serious social and economic problems around the world. According to the U.S. National Survey on Drug Use and Health, 5.2% of pregnant women ages 15 to 44 used illicit drugs in 2006-2007 [1]. In the United States, the average rate of illicit drug use increased slightly from 3.9% in 2004-2005 to 5.2% in 2006-2007. The U.S. study indicates that illicit drug use during pregnancy is a growing problem. In opioid addiction, children born to heroin- or morphine-addicted mothers have been known to suffer from higher mortality and deficiency in the central nerve system [2,3]. Those children may present long-term neuropsychological sequel caused by dysfunction in intellectual ability and in emotional control during their school years [4-6]. These findings underscore the importance of investigating the effects of prenatal opioid exposure in offspring.

Methadone is a synthetic μ-opioid receptor agonist; it is also an antagonist for *N*-methyl-D-aspartate (NMDA) receptor, which is based on its racemic structure [7]. Methadone is commonly utilized in detoxification and maintenance programs for heroin-addicted patients, including pregnant women [8-10]. Methadone maintenance treatment for heroin addicted mothers had been reported to result in lower maternal morbidity/mortality rates and to promote fetal stability and growth, as compared with pregnant women not under methadone maintenance treatment [8,10]. However, high doses of methadone have been found to cause higher neonatal abstinence syndrome (NAS) in offspring [11], suggesting that methadone is not ideal to treat pregnant opioid addicts.

Buprenorphine is a well-established opioid analgesic that recently has been used to treat heroin addiction. Buprenorphine shows complex interactions with various opioid receptor subtypes. It has high affinity to μ- and κ-opioid receptors and also binds to ORL-1 (opioid receptor-like 1) receptor [12,13]. Mu- and κ-opioid and ORL-1 receptor are all expressed in the central nerve system during early prenatal development, hence the use of opioids may affect these receptors during the prenatal period. Recent studies show that buprenorphine maintenance, a new approach to treat heroin dependence, has a lower risk of neonatal abstinence syndrome than methadone [14,15], suggesting that buprenorphine is safer than methadone to treat opioid-addicted women during pregnancy. However, animal studies showed that prenatal exposure to higher dose (1 mg/kg) of buprenorphine affected the myelination in the developing brain [16], indicating that opioid signals played an important role in regulating the brain development of innervations, especially in neuronal axons. The long-term effects of buprenorphine treatment during pregnancy in offspring await further investigation.

Tolerance, the progressive diminution of the susceptibility to the effects of a drug, is an important phenomenon that occurs after chronic opioid administration. Tolerance to morphine-induced analgesia has been found in prenatally morphine-exposed offspring [17-19]. Yet, more studies are needed to investigate tolerance or cross-tolerance development after prenatal exposure to maintenance drugs such as methadone and buprenorphine.

Therefore, we aimed to investigate if the prenatal administration of opioids altered antinociceptive effects of supraspinal analgesia induced by postnatal systemic morphine, methadone, or buprenorphine. The results demonstrate that prenatal administration of morphine, methadone, and buprenorphine brought about the development of a cross tolerance to morphine in the offspring of rats.

## Methods

### Animals

Pregnant Sprague-Dawley rats (BioLASCO Taiwan Co., Ltd) and their offspring were used in the experiments. After arrival, the dams were acclimatized to a room with controlled temperature (25°C), humidity (50 ± 10%) and a 12-h day-night cycle (light on 07:00-19:00 h) for 24 hours before experimentation. Pregnant rats were kept individually in separate cages, and their offspring were housed 2-3 per cage after weaning. All animals were provided with food (Western Lab 7001, Orange, CA, USA) and water *ad libitum*. The ethical guidelines provided by Laboratory Animal Center of the National Health Research Institutes were followed throughout the study.

### Drugs

Morphine (NBCD, Taiwan), methadone (USP, USA), and buprenorphine (Sigma Aldrich, USA) were dissolved in distilled water and were administrated subcutaneously (*s.c.*) in a volume of 1.0 ml/kg of body weight.

Heroin is a major drug of abuse by addicts, however, it is rapidly converted to morphine after crossing the blood brain barrier into the central nervous system. Accordingly, we used morphine directly as a test agent in this study.

### Prenatal treatments

Pregnant Sprague-Dawley female rats, 10-12 weeks old and weighing 200-250 g, were randomly assigned to different groups and were *s.c. *injected with opioids or vehicle during the gestational period (E3 to E20). The dose of opioids used in pregnant rats was selected based on the studies reported previously [17,20]. The treatment protocols for these groups are as follows. Group 1 (vehicle control) rats received 1X phosphate buffer saline 1 ml/kg, *s.c.*, twice a day from E3 to E20. Group 2 (morphine) rats received morphine, 2 mg/kg (initial dose), *s.c.*, twice a day in the first week; the dose was increased by 1 mg/kg every week until the final dose reached 4 mg/kg. Group 3 (methadone) rats received methadone, 7 mg/kg, *s.c.*, twice a day from E3 to E20. Group 4 (buprenorphine) rats received buprenorphine, 3 mg/kg, *s.c.*, once a day from E3 to E20. The offspring were weaned at postnatal day 28 and were maintained until use. The animals at the time of the experiments were 8-12 weeks old with body weight between 250 and 350 g.

### Drug injection protocols

To measure antinociceptive effects of morphine on offspring prenatally exposed to morphine, methadone, and buprenorphine, rats were administrated morphine, 10 mg/kg, *s.c.*, and subjected to the tail-flick test. Rats were treated with morphine twice a day (9:00 and 17:00), and the morphine-induced antinociception was measured after the first injection of morphine every day. To investigate antinociceptive effects of methadone on prenatally methadone-exposed offspring, the testing dose of methadone was 5 mg/kg. Although methadone has a longer duration of action than morphine in humans, its half-life is similar to morphine (70-90 minutes) in rats [21]. In this test, the methadone injection protocol was similar to the morphine protocol as described above. To measure antinociceptive effects of buprenorphine on the prenatally buprenorphine-exposed offspring, rats were injected with buprenorphine, 1.5 mg/kg, *s.c.*, and underwent the analgesic test [22]. Buprenorphine has longer duration of action than morphine and methadone in rats; hence, they were injected with buprenorphine only once a day.

### Analgesia Test

The tail flick test was carried out on rats using a modified method of Dai et al. [23]. The tail flick latency was defined by the time (seconds) the animal withdrew the tail from a heat source (bulb, 8 V/50 W, OSRAM, Germany), and was measured using a semiautomated machine (Model 7369, Ugo Basile, Italy). The infrared intensity of the tail-flick machine was set at 45, which produced a baseline tail flick latency of 2-3 seconds and the cut-off time was set as 10 sec to prevent tissue damage. The rat was put in a restrainer for 5 min for adaption before the tail-flick test was performed. To measure the analgesic effect of opioid agonists, animals were subjected to the tail-flick procedure once a day to minimize the learning effects. All experimental animals were randomly selected from different litters to ensure a general effect in the population. The antinociceptive effects were presented as the area under the time-response curve (AUC = latency × time).

### Data analyses and statistics

All data were analyzed using GraphPad Prism software. Results were expressed as mean ± SEM. Behavioral data were analyzed by an unpaired Student's *t*-test, linear regression, and one-way or two-way ANOVA followed by post-hoc Tukey's multiple comparison. A *P *value < 0.05 was considered significant.

## Results

### Prenatal effects of opioids on the offspring

Results showed that administration of all three opioids (full μ-receptor agonist-morphine/methadone and partial agonist-buprenorphine) decreased the total body weight gain from E3 to E20 in dams. Though the body weight significantly decreased in dams after chronic opioid administration, the average number of pups per litter and the average body weight of the offspring on the first day of birth did not differ significantly from the saline controls. One week after birth, the body weight of the offspring showed a lower increase in prenatally buprenorphine-exposed rats. This phenomenon, however, did not occur in adulthood (8-12 weeks) (data not shown). There was no difference in the fatality of neonatal rats between the saline and morphine/methadone groups; fatality, however, was significantly higher in the prenatally buprenorphine-exposed group than in the saline controls. Fatality among the offspring at P2-P10 of the prenatally buprenorphine-exposed group was also significantly higher than the morphine or methadone prenatally exposed group and saline controls. The results reveal that opioid administration caused changes in weight and neonatal mortality, especially for prenatal exposure to buprenorphine. Effects of prenatal opioid administration on the gross observations of the offspring are summarized in Table 1.

**Table 1:** Effects of prenatal exposure to opioids on offspring

	Saline	Morphine	Methadone	Buprenorphine
	
		Mean ± SEM		
Number of offspring per litter	10.9 ± 0.2	10.5 ± 0.3	9.8 ± 0.3	10.2 ± 0.3
Fatality (%)	0.69 ± 0.33	0	0	7.1 ± 2.38**
Fatality occurred in the offspring (%) (P2-P10)	0.21 ± 0.14	0	0.56 ± 0.56	12.14 ± 7.02*
Body weight increase in the dams (g) (E3-E20)	148.1 ± 2.7	132.3 ± 4.2**	121.3 ± 3.3***	136.4 ± 3.7*
Body weight of the offspring at birth (g)	6.8 ± 0.1	7 ± 0.1	6.6 ± 0.1	6.9 ± 0.1
Body weight of the offspring on day 7 (g)	14.7 ± 0.3	16.3 ± 0.5*	14.5 ± 0.4	13.5 ± 0.4*

*Significantly different compared to saline group, *p < 0.05*

**Significantly different compared to saline group, *p < 0.01*

***Significantly different compared to saline group, *p < 0.001*

### Effects of prenatal morphine administration on morphine-induced supraspinal antinociception

**Figure 1:**
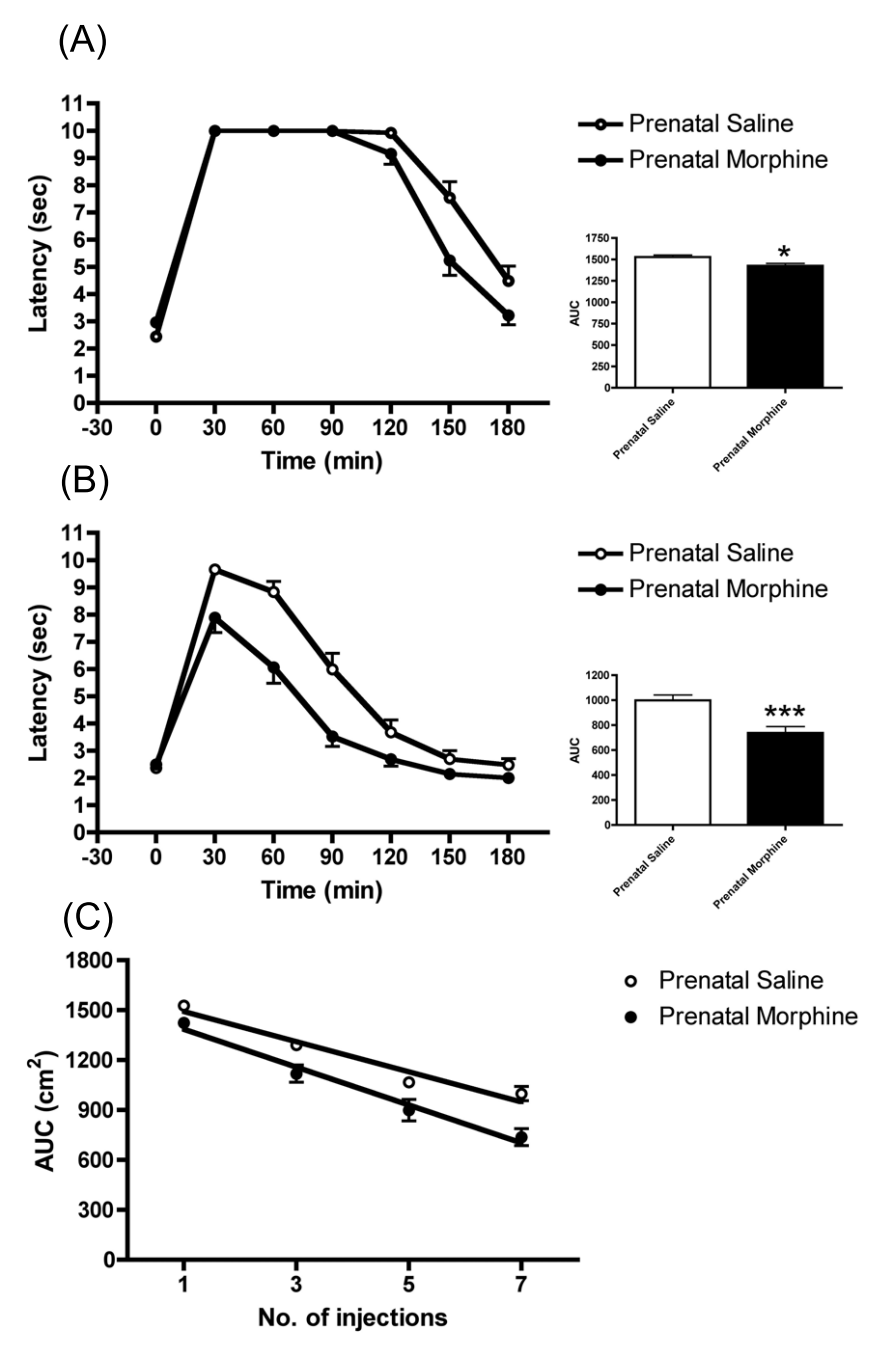
**Tolerance development to morphine in prenatally morphine-exposed male rats**. (A) The latency and AUC of rats after receiving first injection of morphine. (B) The latency and AUC of rats after receiving 7th injection of morphine. (C) Rate of tolerance development to morphine in morphine or saline prenatally exposed rats. The animals more quickly developed a tolerance to morphine than the prenatally saline-exposed controls (F_(1, 114) _= 4.333, p < 0.05). All data are expressed as mean ± S.E.M, (N = 19 per group), *p < 0.05, ***p < 0.001 compared to saline control.

There was a significant decrease of the antinociceptive activity in prenatally morphine-exposed rats in comparison with the prenatal saline controls after the first injection of morphine (Figure 1A). Daily administration of morphine resulted in tolerance development in rats. At the 7th systemic injection of morphine, antinociceptive activity was significantly different between the prenatally saline- and morphine-exposed offspring, with the latter group showing remarkably fewer antinociceptive effects than the saline controls (Figure 1B). The daily recording of the antinociceptive response to morphine revealed that the prenatally morphine-exposed offspring developed a tolerance to morphine more quickly than the saline group (F_(1, 114) _= 4.333, p < 0.05) (Figure 1C). Female offspring exhibited results similar to those of the male offspring in morphine-induced antinociceptive effects (data not shown). These results indicate that prenatally morphine-exposed animals developed a tolerance to morphine more quickly after multiple systemic morphine injections.

### Effects of prenatal methadone administration on methadone-induced supraspinal antinociception

**Figure 2:**
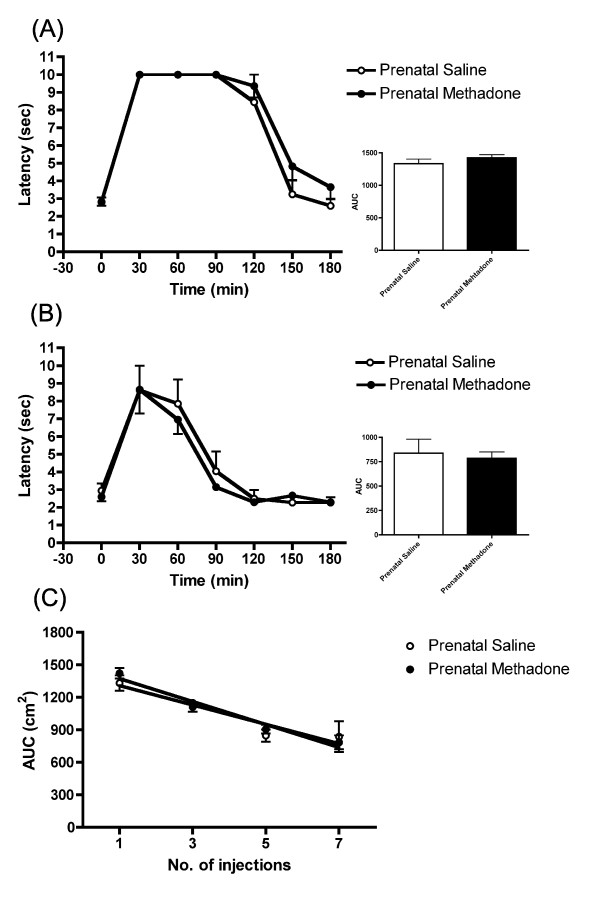
**Tolerance development to methadone in prenatally methadone-exposed male rats**. (A) The latency and AUC of rats after receiving first injection of methadone. (B) The latency and AUC of rats after receiving 7th injection of methadone. (C) Rate of tolerance development to methadone in methadone or saline prenatally exposed rats. There was no difference in tolerance development to methadone (F_(1, 31) _= 0.535, p = 0.471) between the methadone and saline prenatally exposed groups. All data are expressed as mean ± S.E.M, (N = 4 per group).

Postnatal acute treatment with methadone did not result in different antinociceptive response between the prenatally methadone-exposed offspring and the saline controls (Figure 2A). Rats in both groups also developed tolerance to methadone after repeated injection of the drug. At the 7th methadone injection, animals exhibited a decreased analgesic effect of methadone; but there was no difference between the prenatally methadone-exposed group and the saline controls (Figure 2B). The analysis of the daily changes in methadone-induced tolerance on the prenatal methadone-exposed offspring showed no difference from the saline controls (F_(1, 31) _= 0.535, p = 0.471) (Figure 2C). In the female offspring, similar results were obtained (data not shown). These results indicate that acute methadone administration produced the same antinociceptive activity in both prenatally methadone-exposed and saline groups. It also shows that the tolerance development to methadone was not altered in prenatally methadone-exposed offspring.

### Effects of prenatal buprenorphine administration on buprenorphine-induced supraspinal antinociception

**Figure 3:**
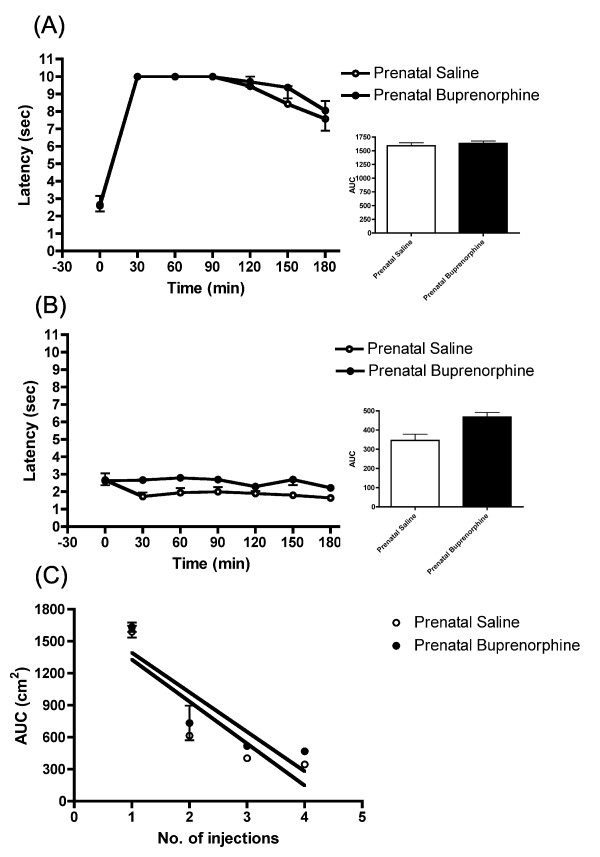
**Tolerance development to buprenorphine in prenatally buprenorphine-exposed male rats**. (A) The latency and AUC of rats after receiving first injection of buprenorphine (B) The latency and AUC of rats after receiving 4th injection of buprenorphine. (C) Rate of tolerance development to buprenorphine in buprenorphine or saline prenatally exposed rats. There was no difference in tolerance development to buprenorphine (F_(1, 31) _= 0.073, p = 0.789) between the buprenorphine and saline prenatally exposed groups. All data are expressed as mean ± S.E.M, (N = 4 per group).

Results showed that postnatal acute injection with buprenorphine did not result in a different antinociceptive response between the prenatally buprenorphine-exposed offspring and the saline controls (Figure 3A). The animals showed a limited antinociceptive response of buprenorphine at the 4th injection of buprenorphine (Figure 3B). In addition, the daily recoding of the data presented a similar development of tolerance between the two groups (F_(1, 31) _= 0.073, p = 0.789) (Figure 3C). The female offspring exhibited similar results (data not shown). The antinociceptive response of buprenorphine showed no difference in the offspring of prenatally exposed buprenorphine and saline controlled group.

### Duration of antinociception in prenatally saline-exposed animals to morphine, methadone, and buprenorphine

**Figure 4:**
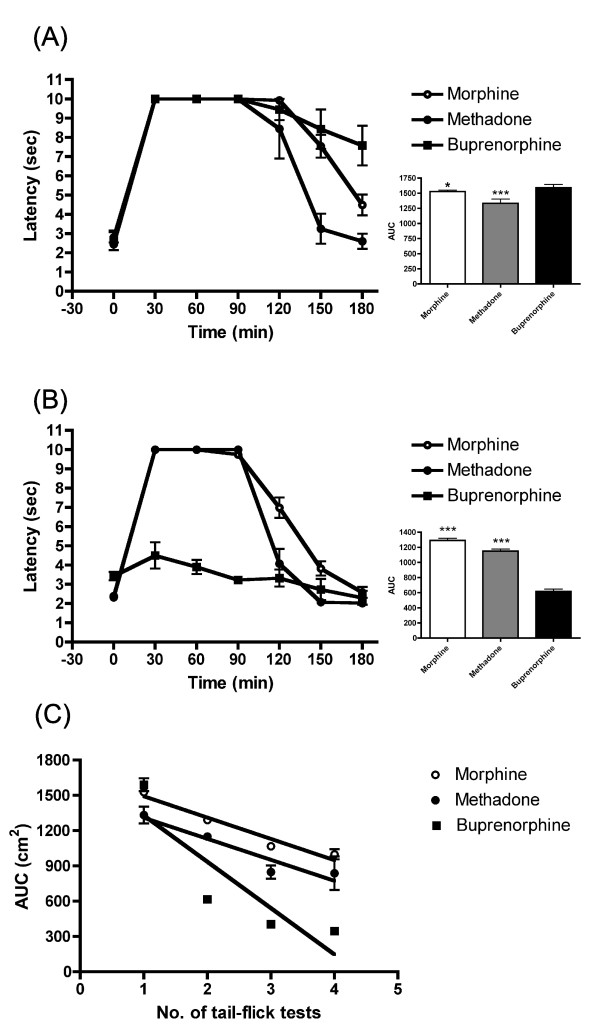
**Antinociceptive response in prenatally saline-exposed male rats after acute or the third (or second) injection of different opioids**. Rats received an acute injection of morphine (10 mg/kg), methadone (5 mg/kg), or buprenorphine (1.5 mg/kg), *s.c.*, at age 8-12 weeks. (A) The latency and the AUC of the first morphine, methadone, or buprenorphine injection. (B) The latency and the AUC of the 3rd morphine and methadone or the 2nd buprenorphine injection. (C) Rate of tolerance development to different opioids in prenatally saline-exposed rats. The rate of tolerance development showed a steeper slope in the buprenorphine-treated group than that of the morphine- or methadone-treated group. All data are expressed as mean ± S.E.M, (N = 4 in methadone and buprenorphine treatment group; N = 19 in morphine treatment group), *p < 0.05, ***p < 0.001 compared to buprenorphine group.

Analyses of the data from the above mentioned experiments on the antinociception in prenatally exposed saline animals are presented in Figure 4. In animals receiving the first injection of buprenorphine, the duration of antinociception was longer than the ones received morphine or methadone (Figure 4A). However, there was no difference in antinociceptive activity between the morphine- and methadone-injected groups (Figure 4A). Furthermore, there was a notable decrease in antinociceptive response after the 2nd administration of buprenorphine, compared with that of the animals receiving the 3rd administration of morphine or methadone (Figure 4B); moreover, the slope of tolerance development was steeper than that of the morphine or methadone group (Figure 4C). These results suggest that acute buprenorphine administration produced better antinociceptive ability than that of the morphine or methadone treated group. In contrast, chronic buprenorphine exposure developed faster tolerance than the other two opioids in rats.

### Effects of prenatal morphine, methadone and buprenorphine administration on morphine-induced supraspinal antinociception

**Figure 5:**
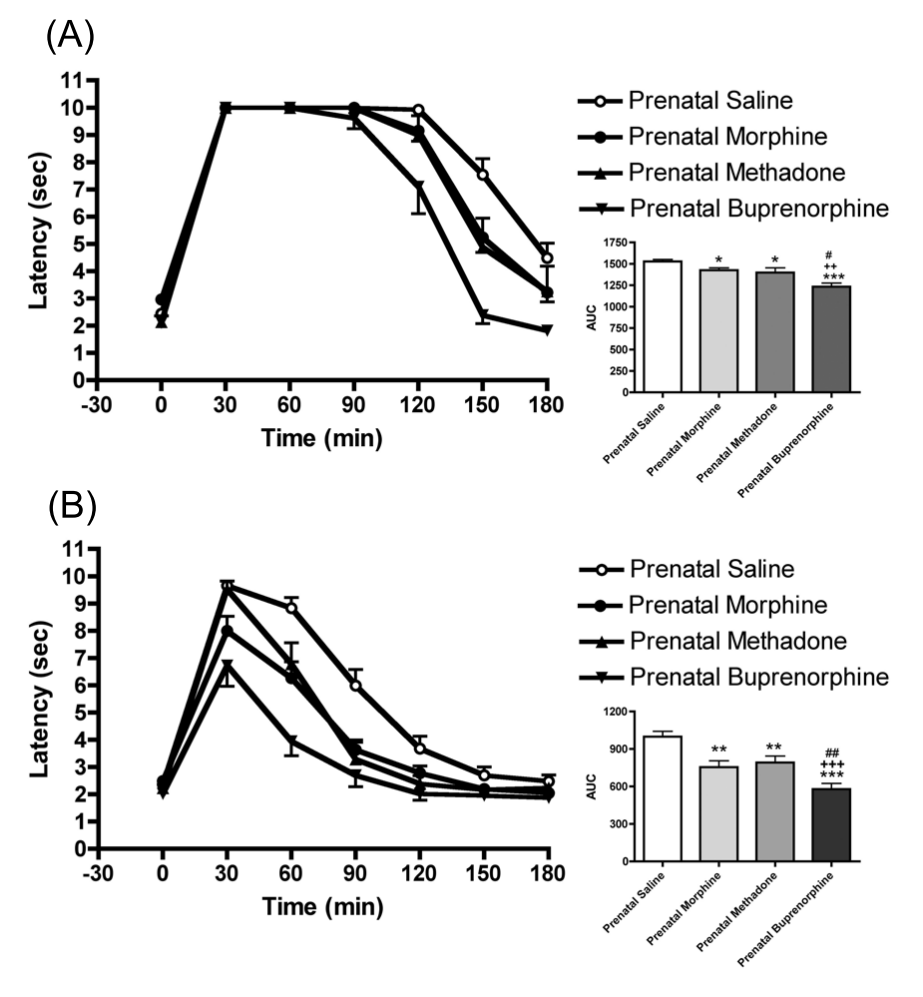
**Cross-tolerance development to morphine in morphine, methadone or buprenorphine prenatally exposed rats**. (A) Tail-flick latency and the area under the curve (AUC) in animals after receiving the first injection of morphine, 10 mg/kg, *s.c. *(B) Tail-flick latency and the AUC in animals after receiving the 7th injection of morphine, 10 mg/kg, *s.c. *All data are expressed as mean ± S.E.M, (N = 9 in prenatally methadone and buprenorphine exposed group; N = 19 in prenatally morphine and saline exposed group), *p < 0.05, ***p < 0.001 compared to saline control; ^++^P < 0.01, ^+++^P < 0.001 compared to morphine; ^#^P < 0.05, ^##^P < 0.01 compared to methadone.

The offspring of all three opioids prenatally exposed rats developed a faster tolerance to morphine. As shown in Figure 5A, the antinociceptive effect was decreased in all prenatally opioid-exposed offspring after acute morphine treatment. Similar analgesic response curves were found in both morphine and methadone prenatally exposed rats. However, buprenorphine prenatally exposed rats were less responsive to morphine-induced antinociception (Figure 5A). All prenatally opioid-exposed groups developed tolerance to morphine after repeated administration of morphine (Figure 5B). The prenatally buprenorphine-exposed group, however, exhibited much less sensitivity to morphine-induced analgesic effects, as compared to morphine or methadone prenatally treated groups (Figure 5B). Comparing the AUC of 1st and 7th morphine administration in different prenatally opioid-exposed rats, these results revealed that the rates (slopes) of tolerance development to morphine in all opioid-exposed groups were faster than the saline control (morphine, F_(1, 71) _= 4.411, p < 0.05; methadone F_(1, 55) _= 14.771, p < 0.001; buprenorphine, F_(1, 55) _= 72.624, p < 0.001). However, the development of tolerance to morphine did not differ between the morphine and methadone prenatally treated groups (F_(1, 54) _= 0.684, p = 0.412). Similar results were also obtained in the female offspring (data not showed). These results indicate a cross-tolerance occurred in the prenatally opioid-exposed offspring after postnatal morphine administration. The prenatally buprenorphine-exposed offspring showed a significantly higher cross-tolerance to morphine than the prenatally morphine- or methadone-exposed offspring.

## Discussion

The goal of this study was to compare effects of prenatal exposure to morphine, methadone, and buprenorphine on offspring when they were re-exposed to opioids at adulthood. Treatments with all these opioids caused weight loss in dams but did not directly affect the birth weight of the offspring. Though the weight of the offspring did not differ on the first postnatal day, the pups in the buprenorphine group showed a significant loss in body weight after one week, that may reflect the potential existence of neonatal abstinence syndrome. During the tail-flick testing period at age 8-12 weeks, there was no difference in the average of body weight in all of the opioid treated groups. Prenatal exposure to morphine enhanced the rate of tolerance development to morphine in the offspring. However, development of tolerance to methadone or buprenorphine in prenatally methadone- or buprenorphine-exposed offspring, respectively, was not observed any difference with prenatally saline exposed controlled group. Furthermore, all prenatally opioids-exposed rats showed faster development of tolerance to morphine. Most notably, the prenatally buprenorphine-exposed group exhibited much less sensitivity to morphine-induced antinociceptive effect, compared to the morphine and methadone prenatally treated groups. The effect of prenatal exposure to opioids that was observed in the behaviors when the animals were challenged with opioid at adulthood indicates that prenatal opioid administration may cause long-term changes in offspring.

The decrease in body weight in opioid-treated dams might be due to less consumption of food [24]. The dose of methadone used was higher than that of morphine in this study to mimic the therapeutic procedure in human. However, a notable catatonic effect was observed in methadone *s.c. *injected dams in comparison with the morphine-injected group (data not shown). The marked catatonic effect of methadone on rats might lead to the lower weight increase in methadone-administrated dams than other groups. Buprenorphine is the newest drug used to treat heroin addicts. Several clinical studies have suggested that buprenorphine is useful for ameliorating neonatal abstinence syndrome in infants [8,14]. Previous studies showed no mortality occurred in prenatal exposure to 3 mg/kg/day of buprenorphine in rats [25]. However, higher mortality in the offspring with the same dose of buprenorphine administrated to dams was observed in our study. Our results agree with those in a previous study by Robinson and Wallace [20], that higher dose (3 mg/kg) of buprenorphine increased the number of stillbirths and raised the mortality index, indicating that higher dose of buprenorphine might induce complex effects or serious systemic toxicity to the offspring.

In agreement with previous reports [17-19], our study found faster decreases in the antinociceptive response to morphine among prenatally morphine-exposed offspring than among the controls. However, some studies obtained opposite results demonstrating that prenatal exposure to morphine enhanced antinociceptive response to morphine in adult rats [24,26]. This discrepancy might be due to different injection schedules (short term with low dose) or measurement methods (hot plate) used in experimental design. Several reports provided possible mechanisms to explain the prenatally morphine-induced tolerance to morphine, including changes in opioid receptor density [17,27,28], intracellular cAMP levels [29], G protein mRNA levels [30], and expression of endogenous opioids [31]. Prenatal morphine exposure increased μ-opioid receptor protein and mRNA expression at P1 and P7; but the expression returned to the normal level at P14 [27]. Other studies [17] also showed that μ-opioid receptor binding of the whole brain homogenate of P14 offspring of rats did not differ between the prenatally saline- and morphine-exposed groups. Nevertheless, autoradiographic study in P14 rats revealed that the density of the μ-opioid receptor was significantly decreased in the striatum, thalamus, and amygdala, but not in the midbrain, nucleus accumbens (NAc), hippocampus, or cortex in the prenatally morphine-exposed offspring [17], suggesting that changes in opioid receptor density in the offspring of morphine prenatally exposed animals are region specific. Evidence available also demonstrates sex-dependent effects in μ-opioid receptor densities in the brain of animals prenatally exposed to morphine. Prenatally morphine-exposed male rats showed an increase in μ-opioid receptor densities in the NAc and posteromedial cortical amygdala, and a decrease in the basolateral amygdala. However, the changes in μ-opioid receptor densities were dependent on ovarian hormones in the female rat [32]. Though the results showed a gender difference in μ-opioid receptor densities, the antinociceptive response to opioids did not reveal gender difference in our study.

We obtained no difference in antinociceptive response to methadone in prenatally saline- and methadone-exposed rats. However, a previous study showed controversial results that prenatal exposure to methadone enhanced antinociceptive response to methadone in adult rats [33]. The difference in these findings of antinociceptive response seems due to different measurement methods for antinociceptive response (hot plate vs. tail flick) and age (120 days vs. 60 days) used in experimental design. It is well known that pharmacologic profiles of methadone are similar to morphine and they also have similar antinociceptive effects by peripheral subcutaneous injection [34]. However, tolerance development to methadone in the prenatally methadone-exposed rats was not observed. This is different from what we obtained in the case of morphine. A possible explanation may be the use of racemic mixture of methadone, which is a μ-opioid receptor agonist and also an N-methyl-D-aspartate (NMDA) antagonist in our study. Davis and Inturrisi [35] used d-methadone to block morphine-induced tolerance and NMDA-induced hyperalgesia. Their results indicate that methadone-induced antinociceptive response may be a net outcome of methadone acting on two different classes of receptors that have opposing regulating functions on opioid-induced antinociception. Their finding may explain why we obtained different results of antinociceptive response and tolerance development to morphine and methadone.

In addition, our study obtained similar antinociceptive response and development of tolerance to morphine in prenatally methadone- and morphine-exposed offspring. The morphine-induced tolerance in prenatally methadone-exposed animals might also be due to opioid receptor expression or binding affinity. Prenatal methadone treatment induced sustained decreases in both δ- and μ-opioid receptors in the hypothalamus but not in the cerebral cortex [36]. A previous study by Darmani et al. [37] did not obtain changes in the expression of μ-receptors when animals were prenatally exposed to methadone, 6.3-9.0 mg/kg/day, from gestation days 7 to 20. However, when animals were chronically prenatally exposed to methadone, μ-opioid receptor affinity in both fetal and maternal brain homogenates at day 20 of the pregnancy was reduced [37]. Darmani et al. [37] also proposed that the effect of prenatal exposure to methadone induced an increase in the Kd of μ-opioid receptor binding as a transient effect that returned to the control value at day 7 after delivery. However, the region specificity of opioid receptor expression remains unclear.

In this study, we also demonstrated that prenatally saline-exposed rats developed tolerance to buprenorphine more rapidly than to morphine or methadone treatment. This confirmed the previous finding that higher tolerance development to the opioid occurred not only in a high-efficacy opioid, morphine, but also in a low-efficacy opioid, buprenorphine [38]. Buprenorphine induced broad and complicated effects in the neural system due to its action on μ-, κ-, δ-, and ORL-1 receptor. The mechanism of faster tolerance development to buprenorphine in prenatally saline-exposed rats may due to higher receptor affinity and slower dissociation of the drug from the receptors [12,38]. Repeated treatment of buprenorphine induced even greater hyperanalgesia than morphine, indicating that chronic buprenorphine treatment may reset the pain threshold [38]. It has been reported that buprenorphine-induced antinociceptive response is via the μ-opioid receptor [39]. Moreover, it also has been shown that morphine or buprenorphine-induced antinociception was significantly reduced in animals after they received chronic administration of buprenorphine [40]. This indicates that a primary factor for buprenorphine to induce faster tolerance development in prenatally saline-exposed rats may be a significant alteration of the μ-opioid receptor after buprenorphine treatment.

We were the first to demostrate that the prenatally buprenorphine-exposed rats at age 8-12 weeks showed faster tolerance development to morphine. Previous study showed that rats prenatally exposed to methadone or to low or high doses of buprenorphine exhibited more resistance to morphine-induced antinociception in 4-day postnatal pups [20]. Robinson et al. [20] also found that morphine ED_25 _values were highly increased in pups prenatally exposed to buprenorphine, compared with methadone after morphine challenge. Methadone increased the ED_25 _of morphine when the pups exposed to methadone in both pre- and postnatal stage. However, pups exposed to buprenorphine either prenatally, postnatally, or both pre- and postnatally were more resistant to the antinociceptive response to morphine. According to this finding, Robinson et al. noted that buprenorphine seemed to have a greater ability than methadone to induce tolerance to morphine and did so in a dose-related manner [20]. However, our results provided direct evidence to show that prenatal exposure to buprenorphine caused faster development of tolerance by daily challenge with morphine, than prenatally saline-, morphine-, and methadone-exposed groups at adulthood. Since buprenorphine is very lipid soluble and dissociated slowly from the receptors [12,38], prenatal buprenorphine administration may remain in the body of the offspring till the postnatal period; hence, it may antagonize the effects of morphine. However, according to our study, that the experiment was conducted at adulthood ruled out the residual effects of buprenorphine in the offspring. Earlier studies suggested that daily repeated exposure to buprenorphine reduced the morphine-induced analgesia in naive rats [38,40,41], suggesting that buprenorphine could produce cross-tolerance to morphine in normal condition. However, the mechanism of the cross-tolerance occurred in the drug-free of opioid prenatally exposed offspring awaits further studies. Possible regulatory mechanism might be associated with changes in opioid or opioid-like receptor densities. Prenatal exposure to a higher dose of buprenorphine caused a reduction in the expression of μ-opioid receptor and an increase of κ-opioid receptor in the brain of P1 offspring [42]. While Belcheva et al. [42,43] speculated a transient effects of burprenorphine that the receptor densities of brain homogenates returned to normal at P7, the differential distributions on specific brain areas at adulthood warrant further studies. Recently, buprenorphine has been shown to act as an agonist on the ORL-1 receptor [12], which is expressed as early as E12 in the cortical plate, basal forebrain, brainstem, and spinal cord, and which may play important roles in maturation of stress and pain circuitry [44]. It is also a type of pain-related receptor that is involved in buprenorphine-induced antinociceptive response [39]. Therefore, buprenorphine may target several classes of receptors during brain development; hence, prenatally administrated buprenorphine may have a greater impact on brain development than other opioids.

## Conclusions

In summary, our results confirmed and extended those of previous studies. We compared three types of opioids in the same analgesic test condition to verify the effect of prenatal exposure to opioids on the offspring at their adulthood. Although methadone and buprenorphine are considered safer substances than morphine for treating heroin addicts, prenatal exposure to opioids has been shown to induce faster tolerance development to morphine. We found that prenatal exposure to buprenorphine showed higher mortality and much less sensitivity to morphine, indicating that buprenorphine in higher doses may not be an ideal maintenance drug for treating pregnant women. Therefore, this study has provided further evidence to show that prenatal effects of opioids on the opioidergic system exhibit long-term changes even at adulthood. Furthermore, several previous studies suggested that the changes of opioid receptors in prenatal exposure to opioids may be the primary factor in behaviors; however, details of the changes of the opioid receptors in different brain regions are still unclear. For this reason, we will further examine region specificity of prenatal opioid exposure on different opioid receptors in rats at their adulthood in a future investigation. Finally, higher doses of buprenorphine caused notably more serious side effects than other opioids in this study, which could provide a reference in selecting doses for clinical usage in treating pregnant women who are heroin addicts.

## Competing interests

The authors declare that they have no competing interests.

## Authors' contributions

YCC designed and performed the experiments, analyzed the data, and drafted the manuscript. TWH co-performed the experiments and participated in the discussion of the experimental results. JYY and CWSL co-performed the experiments. IKH conceived the study, coordinated its implementation, and revised the final manuscript. All authors read and approved the final manuscript.
